# High Neutrophil to Lymphocyte Ratio and Its Gene Signatures Correlate With Diastolic Dysfunction in Heart Failure With Preserved Ejection Fraction

**DOI:** 10.3389/fcvm.2021.614757

**Published:** 2021-06-24

**Authors:** Bo Bai, Min Cheng, Lingyan Jiang, Jiabin Xu, Haibo Chen, Yun Xu

**Affiliations:** Department of Cardiology, The First Affiliated Hospital of Shenzhen University, Shenzhen Second People's Hospital, Shenzhen, China

**Keywords:** heart failure with preserved ejection fraction, neutrophil to lymphocyte ratio, inflammation, diastolic dysfunction, gene signature

## Abstract

**Aims:** To evaluate the interrelation between neutrophil to lymphocyte ratio (NLR) coupled with gene signatures, inflammation, and diastolic dysfunction in patients with heart failure (HF) with preserved ejection fraction (HFpEF).

**Methods:** The clinical profile of 172 patients with HFpEF (EF ≥ 50%) and 173 non-HF control individuals was analyzed retrospectively. The association between NLR and HFpEF and the predictive performance of NLR for HFpEF were assessed by the binary logistic regression analysis and the receiver operating characteristic curve (ROC). Multivariate linear regression models further examined the associations between NLR and high-sensitivity C-reactive protein (hs-CRP), N-terminal prohormone of brain natriuretic peptide (NT-proBNP), and average septal-lateral E/e', respectively. The freshly isolated neutrophils from 30 HFpEF patients and 42 non-HF controls were subjected to transcriptomic profiling. The biomarkers related to neutrophil activation and inflammation were detected in serum samples.

**Results:** The HFpEF patients in Southeast China were lean and had comorbidity burden and worse cardiac structure/function. Compared with non-HF control individuals, HFpEF patients had a rise in NLR. NLR displayed an independent association with HFpEF [adjusted odds ratio, 2.351; 95% CI, 1.464–3.776; *p* < 0.001] and it predicted HFpEF with the area under the ROC 0.796 (95% CI, 0.748–0.845, *p* < 0.001). The positive associations between NLR and hs-CRP, NT-proBNP, and mitral E/e' were found in HFpEF patients. Moreover, patients had significantly elevated serum levels of neutrophil elastase and inflammatory biomarkers, both of which correlated with the mitral E/e' ratio. Finally, multiple molecules that drive neutrophil degranulation and inflammation, such as *S100A8*/*A9*/*A12* and *PADI4*, were transcriptionally up-regulated in neutrophils of HFpEF patients.

**Conclusions:** The high NLR coupled with transcriptional activation of neutrophils correlates with systemic inflammation and functional impairment in HFpEF patients, which may suggest a causative role of neutrophils in the pathogenesis of the disease.

## Introduction

Heart failure (HF) with preserved ejection fraction (HFpEF) has conferred substantial morbidity and mortality on clinical patients. Its prevalence is increasing at an alarming rate, currently representing 50% of all HF worldwide (1). In contrast to positive outcomes in heart failure with reduced ejection fraction (HFrEF) treated by neurohumoral inhibition, most of the large-scale clinical trials to assess the efficiency of medical therapies for HFpEF have not shown positive results yet (2, 3). HFpEF represents a broad cohort of patients with a combination of multiple risk factors and comorbidities. As such, the failure of effective treatment for HFpEF is likely attributable to the heterogeneity in this clinical scenario (4). Despite the phenotypic diversity, an increasingly popular theory about HFpEF is that this syndrome reflects a pro-inflammatory state (5). By utilizing comprehensive proteomic approaches to analyze blood biomarkers of HFpEF patients, recent studies demonstrate that systemic inflammation is closely related to HFpEF symptomatology. Moreover, the inflammation appears to mediate the association between comorbidity burden, worse cardiac hemodynamic stress, and adverse outcomes (6, 7). The systemic inflammation is associated with increased cardiomyocyte passive tension and aberrant myocardial collagen deposition, both of which would result in impaired left ventricular (LV) compliance in HFpEF (5, 8). Intriguingly, the tissue or cellular source of these inflammatory biomarkers remains uncertain. Therefore, characterizing specific sources of inflammatory molecules involved in the pathogenesis of HFpEF is an essential issue to be clarified.

Neutrophils are the dominant type of leukocytes during acute inflammatory reactions. The emerging evidence that neutrophils contribute to the clinical manifestations of cardiovascular diseases has been well-discussed (9). In the context of congestive HF, the increased neutrophil lifespan positively correlates to the New York Heart Association (NYHA) class, plasma levels of C-reactive protein (CRP), and alkaline phosphatase (10). The I-PRESERVE trial (Irbesartan in Heart Failure with Preserved Ejection Fraction Study) demonstrates that high neutrophil counts serve as an independent risk factor associated with poor outcomes of HFpEF patients (11). The neutrophil to lymphocyte ratio (NLR) has been proposed as a valuable marker to stratify the risk of patients hospitalized with HFpEF (12). The high level of plasma myeloperoxidase secreted by neutrophils is thought to be suggestive footprints of microvascular endothelial inflammation in HFpEF patients (13). In the endomyocardial biopsy samples from HFpEF patients, a subset of inflammatory cells marked by CD11a and CD45 (pan-leukocyte markers) is increased, associated with the collagen accumulation and high tissue levels of transforming growth factor (TGF)-β (14). Our previous study reported a lean diabetic HFpEF mouse model. The HFpEF mice have diastolic dysfunction and LV stiffness, concurrent with apparent cardiac inflammation and interstitial fibrosis. Of note, these pathological alternations in mouse hearts are associated with massive neutrophil infiltration and neutrophil extracellular traps (NETs) formation (15).

It appears that neutrophils play significant roles in the pathogenic process of HFpEF. However, the pathological involvement of neutrophils in exacerbating the inflammation or functional impairment of HFpEF patients remains poorly understood. To this end, our study aimed to assess the interrelation between neutrophils coupled with transcriptomic profile, inflammatory biomarkers, and abnormal cardiac structure/function of clinical HFpEF patients.

## Methods

### Study Population

First, in retrospective analysis, the clinical data were obtained from 172 in-patients diagnosed with HFpEF (EF ≥50%) between January 2016 and December 2019 (16). Meanwhile, 173 gender and age-matched in-patients with mild to moderate hypertension but no HF symptoms were recruited as non-HF controls (27 patients had Grade1 hypertension, 144 patients had Grade 2 hypertension, and two patients had Grade 3 hypertension) (Table 1). Patients' clinical profile, including demographic variables, medical history, laboratory values, and echocardiographic variables, was well-documented after admission. Patients who had pulmonary infection, hematopoietic disease, and autoimmune disease or were undergoing antibiotic or immunosuppressive therapy were excluded from this study. Second, a total of 30 in-patients newly diagnosed with HFpEF and 42 non-HF control individuals in our hospital (from January to December 2020) were enrolled in this study (Supplementary Table 1). The circulating neutrophils were freshly isolated from blood samples of patients for transcriptomic analysis. The serum samples of patients were subjected to a biomarker assay.

**Table 1:** The clinical characteristics of non-HF control individuals and HFpEF patients.

	**Non-HF**	**HFpEF**	***p*-Value**
	**(*n =* 173)**	**(*n =* 172)**	
**Demographic characteristics**			
Age	70.8 ± 5.8	71.1 ± 12.5	0.24
Female	82 (47.6)	83 (48.3)	0.91
BMI, kg/m^2^	24.7 ± 3.2	23.7 ± 4.5	< 0.05
Heart rate, beats/min	73.9 ± 13.1	84.3 ± 19.8	< 0.05
Systolic BP, mm Hg	138.6 ± 16.5	133.1 ± 21.8	< 0.05
Diastolic BP, mm Hg	80.9 ± 10.9	77.5 ± 15.5	< 0.05
**Medical history**			
NYHA functional class			< 0.05
I	5 (2.9)	2 (1.2)	
II	2 (1.2)	14 (8.1)	
III	0 (0)	41 (23.8)	
IV	0 (0)	115 (66.9)	
Hypertension	173 (100)	123 (71.5)	< 0.05
Diabetes mellitus	31 (17.9)	58 (33.7)	< 0.05
Hyperlipidemia	48 (27.7)	20 (11.6)	< 0.05
Arrhythmia	33 (19.1)	111 (64.5)	< 0.05
Coronary vascular disease	14 (8.1)	89 (51.7)	< 0.05
**Medication use**			
Antiplatelet therapy	120 (69.3)	160 (93.0)	< 0.05
Beta-blockers	71 (41.0)	150 (87.2)	< 0.05
Calcium-channel blockers	117 (67.6)	39 (22.7)	< 0.05
Diuretics	11 (6.4)	170 (98.8)	< 0.05
ACE inhibitors or ARBs	95 (54.9)	124 (72.1)	< 0.05
Statins	157 (90.8)	138 (80.2)	< 0.05
**Echocardiography**			
LV mass, g	165 (139, 186)	253 (211, 315)	< 0.05
LVEF, %	67 (65, 70)	61 (55, 65)	< 0.05
E/e'	11.4 (8.9, 13.4)	18.5 (14.5, 26.2)	< 0.05
LA diameter, cm	3.2 (3.0, 3.4)	4.2 (3.9, 4.7)	< 0.05
**Laboratory**			
NT-proBNP, pg/ml	88 (53, 156)	3,320 (1,657, 7,991)	< 0.05
hs-CTnI, ng/ml	0.01 (0.01, 0.02)	0.01 (0.01, 0.02)	0.27
Creatinine, μmol/L	65.5 (53.8, 76.6)	95.0 (71.6, 144.7)	< 0.05
Total triglyceride, mmol/L	1.24 (0.95, 1.74)	0.94 (0.77, 1.55)	< 0.05
Total cholesterol, mmol/L	4.27 (3.67, 4.98)	3.67 (3.16, 4.47)	< 0.05
LDL-C, mmol/L	2.64 (2.05, 3.29)	2.08 (1.70, 2.79)	< 0.05
HDL-C, mmol/L	1.22 (1.05, 1.39)	0.99 (0.82, 1.26)	< 0.05
Fasting Glucose, mmol/L	5.15 (4.79, 5.73)	5.38 (4.72, 6.84)	< 0.05
HbA1c, %	5.8 (5.5, 6.1)	6.1 (5.7, 6.7)	< 0.05
hs-CRP, mg/L	1.50 (0.48, 2.73)	9.62 (3.03, 27.38)	< 0.05
**Hematological parameters**			
WBC count, 10^9^/L	6.19 (5.23, 7.29)	6.79 (5.54, 8.96)	< 0.05
RBC count, 10^12^/L	4.38 (4.04, 4.71)	4.10 (3.60, 4.62)	< 0.05
Platelet count, 10^9^/L	209 (178, 244)	198 (158, 248)	0.19
Hemoglobin, g/L	132 (124, 143)	119 (101, 136)	< 0.05
Neutrophil, 10^9^/L	3.79 (3.05, 4.48)	4.71 (3.58, 6.74)	< 0.05
Lymphocyte, 10^9^/L	1.79 (1.40, 2.16)	1.29 (0.92, 1.74)	< 0.05
Monocyte, 10^9^/L	0.49 (0.37, 0.61)	0.50 (0.33, 0.67)	0.24
NLR	2.21 (1.60, 2.74)	3.77 (2.43, 5.76)	< 0.05

*Data are given as mean (SD), median (IQR), or number (percent), as appropriate*.

*Depending on the types of data, the Mann-Whitney test or Fisher exact test for unpaired observations was applied, and p < 0.05 was considered to indicate statistical significance*.

*ACEI, angiotensin-converting enzyme inhibitors; ARBs, angiotensin receptor blockers; BMI, body mass index; BP, blood pressure; E/e', average septal-lateral E/e' ratio; HbA1c, hemoglobin A1c; HDL-C, high-density lipoprotein cholesterol; hs-CTnl, high-sensitivity cardiac troponin I; hs-CRP, high-sensitivity C-reactive protein; LA, left atrial; LDL-C, low-density lipoprotein cholesterol; LVEF, left ventricular ejection fraction; LV mass, left ventricular mass; NT-proBNP, N-terminal prohormone of brain natriuretic peptide; NYHA, New York Heart Association; RBC, red blood cell; WBC, white blood cell; NLR, neutrophil count to lymphocyte count*.

### Assessment of Hematological Parameters

Laboratory variables, including complete blood cell counts, serum lipids, glucose, hemoglobin A1c (HbA1c), N-terminal prohormone of brain natriuretic peptide (NT-proBNP), were examined and documented. The NLR ratio was constructed as follows: NLR = neutrophil count to lymphocyte count.

### Neutrophil Isolation

The circulating neutrophils were freshly isolated from eight HFpEF patients and 12 non-HF control individuals. The blood sample (2 mL) was carefully layered over Polymorphprep™ reagent (Axis-Shield, Scotland). After centrifuging at 500 g for 35 min at room temperature, the plasma and mononuclear cells (upper band of cells) were removed, and neutrophils were harvested. After washing with Hepes-buffered saline [0.85% (w/v) NaCl], cell pellet was resuspended in ammonium chloride lysis buffer [0.83% (w/v) NH4Cl, 10 mM Hepes-NaOH, pH 7.4] to remove any residual erythrocyte contamination. Then cells were harvested by centrifugation and stored in TRIzol™ reagent (Thermo Fisher Scientific, USA) for subsequent RNA extraction.

### Transcriptome Sequencing of Neutrophils

RNA isolation and purification were performed using TRIzol-chloroform and RNeasy Mini Kit (Qiagen, Germany). The quality of RNA was checked with Agilent 2100 Bioanalyzer (Agilent, USA). The RNA-seq library was prepared by the Beijing Genomics Institute (Shenzhen, China). Sequence reads were obtained using BGIseq500 (Illumina) and successfully mapped to the human genome (Genome Reference Consortium Human Build 38 patch release 13, GRCh38.p13). Read counts were normalized based on reads per kilobase million (RPKM). The DEseq2 method was used to filter differential genes (17). The adjusted *p*-value (Q value) < = 0.05 was acceptable to indicate the gene expression with a significant difference. According to the results of differential gene detection, the R package heatmap was used to perform hierarchical clustering analysis on the union set differential genes. The Reactome enrichment was subsequently performed to investigate the molecular function and biological pathways that genes participate.

### Determination of Biomarkers in Serum

The serum samples were collected from 30 HFpEF patients and 42 non-HF control individuals. Biomarkers related to systemic inflammation [interleukin (IL)-1β, IL-6, IL-10, tumor necrosis factor (TNFα), and soluble intercellular adhesion molecule-1 (sICAM-1)], tissue remodeling [matrix metalloproteinase 9 (MMP9)], as well as neutrophil activation [neutrophil elastase (NE)] were examined by the enzyme-linked immunosorbent assay (ELISA) according to the manufacturer's instructions (NeoBioscience, China, and Abcam, UK).

### Statistical Analysis

In the cohort study, data are given as means and standard deviations (SD), medians and interquartile 25th and 75th percentiles (IQRs), or numbers and percentages, as appropriate. The statistical calculations were performed using IBM SPSS statistics software. Depending on the types of data, the non-parametric Mann-Whitney test or Fisher exact test for unpaired observations was applied for statistical comparison. Binary logistic regression analysis was carried out using HFpEF as the dependent variable to analyze the association between NLR and HFpEF after adjusting for potential confounders, including age, gender, body mass index (BMI), high-sensitivity CRP (hs-CRP), and diabetes. The crude and adjusted odds ratios (OR) with 95% confidence intervals (CI) were calculated. Receiver operating characteristic (ROC) curves with area under the curve (AUC) were calculated to determine discriminative ability. The partial Pearson or the Spearman correlations were computed to describe the relationship between variables of interest after values were logarithmically transformed. Subsequently, the multivariate linear regression analysis was conducted to identify factors associated with hs-CRP, NT-proBNP, and average septal-lateral E/e', respectively. In the transcriptomic sequencing study, the fold changes of FPKM of interested genes were calculated and compared between the two groups. Independent samples were compared by a two-tailed unpaired *t-*test with Welch's correction. For all statistical analyses, *p* < 0.05 was considered to indicate statistical significance.

## Results

### The Clinical Characteristics of HFpEF Patients

Baseline demographic characteristics and laboratory variables of the entire study population were shown in Table 1. The age and gender distribution of HFpEF patients were comparable to that of the non-HF control population. Of note, both groups of cohorts were lean and with an average BMI below 30 kg/m^2^. The majority of HFpEF patients had severe cardiac function impairment. Approximately 90% of patients were classified in NYHA III to IV, together with a significant elevation of NT-proBNP in patients' blood. Besides hypertension, comorbid arrhythmia and coronary vascular disease were frequently present in HFpEF patients. Of note, we found diabetes was more common in the HFpEF group (30.8 *vs*.12.7% of cohorts who had HbA1c>6.5% for HFpEF *vs*. non-HF individuals). Compared with non-HF individuals, a greater proportion of HFpEF patients were on an antiplatelet, beta-blocker, diuretic, and angiotensin-converting enzyme inhibitors (ACEI)/angiotensin receptor blockers (ARBs) therapy. Echocardiography examination demonstrated that HFpEF patients had an overall prevalence of LV hypertrophy. Although HFpEF patients exhibited a preserved LV ejection fraction (LVEF, 55–65%), the significantly increased average septal-lateral E/e' ratio, one of the echocardiographic markers of LV filling pressure (18), was present among patients. Moreover, HFpEF patients exhibited an apparent left atrial (LA) dilatation compared to non-HF controls. In terms of laboratory variables, HFpEF patients had significantly elevated hs-CRP levels in circulation, underpinning a systemic inflammatory state in patients.

### Correlations Between NLR, Inflammation, and Echo Characteristic

**Figure 1:**
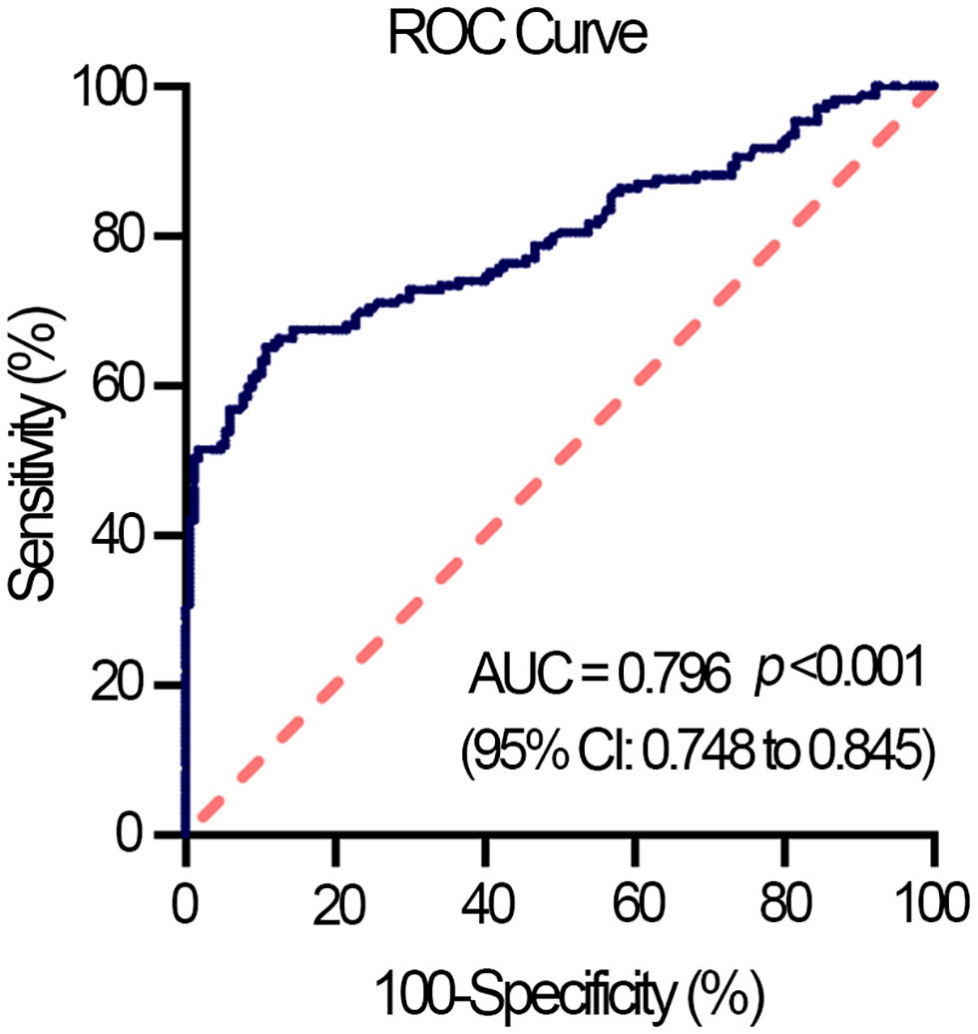
Receiver operating characteristic (ROC) curve displays NLR as a predictor of HFpEF in the entire study population (*n* = 345). AUC, the area under the curve; CI, confidence interval; HFpEF, heart failure with preserved ejection fraction.

The total count of leukocyte, red blood cells, platelet, and monocytes of HFpEF patients was within normal range and was not distinctly different from that of non-HF controls. However, HFpEF patients had a higher neutrophil count but lower lymphocyte count than that of non-HF controls, which resulted in a significant rise in NLR of HFpEF patients (Table 1). The binary logistic regression analysis showed that NLR was significantly associated with HFpEF, independent of effects of age, gender, BMI, hs-CRP, and diabetes (adjusted OR, 2.351; 95% CI, 1.464–3.776; *p* < 0.001) (Table 2). Then, we calculated the AUC in the ROC curve to assess the predictive performance of NLR for HFpEF, which was 0.796 [95% CI (0.748–0.845), *p* < 0.001] (Figure 1).

**Table 2:** Binary logistic regression analysis of factors associated with HFpEF.

**Model**	**Variables**	**OR**	**95% CI**	***p***
Crude model	NLR	2.626	(2.036 to 3.386)	< 0.001
Adjusted model	NLR	2.351	(1.464 to 3.776)	< 0.001
	Age	0.961	(0.910 to 1.015)	0.152
	Female	1.133	(0.453 to 2.835)	0.789
	BMI	0.899	(0.791 to 1.021)	0.102
	hs-CRP	1.719	(1.334 to 2.215)	< 0.001
	Diabetes	1.319	(0.447 to 3.088)	0.616

*Binary logistic regression analysis was applied to examine factors associated with the HFpEF (dependent variable) in the entire study population (n = 345). The p < 0.05 was considered to indicate statistical significance. OR, odds ratio; CI, confidence interval. Other abbreviations as in Table 1*.

The significant correlations between NLR and hs-CRP, NT-proBNP, and mitral E/e' ratio were found in HFpEF patients, which were not changed after adjusting age, gender, BMI, NYHA class, and diabetes (Supplementary Table 1). Furthermore, results of multivariate linear regression models suggested NLR was likely to be an independent predictor of hs-CRP (*p* < 0.001), NT-proBNP (*p* < 0.01), and mitral E/e' ratio (*p* < 0.05), respectively (Table 3). In contrast, NLR did not display any significant correlation with cardiac structural parameters in HFpEF patients (Supplementary Table 1).

**Table 3:** Multivariate linear regression analysis with hs-CRP, NT-proBNP, and E/e' as dependent variables, respectively.

**Regression variables**	***B***	**VIF**	**95%CI**	***p***	***R^**2**^***	P*
**hs-CRP**					0.164	< 0.01
Constant term	−0.010					
NLR	0.677	1.095	(0.286 to 1.067)	< 0.001		
Age	0.001	1.140	(−0.009 to 0.010)	0.908		
Female	−0.224	1.145	(−0.469 to 0.021)	0.730		
BMI	0.015	1.102	(−0.011 to 0.042)	0.250		
NYHA class	0.053	1.263	(−0.128 to 0.234)	0.562		
Diabetes	0.149	1.116	(−0.111 to 0.409)	0.258		
**NT-proBNP**					0.197	< 0.001
Constant term	2.517					
NLR	0.514	1.107	(0.186 to 0.843)	< 0.01		
Age	0.003	1.165	(−0.004 to 0.010)	0.358		
Female	−0.032	1.156	(−0.218 to 0.153)	0.731		
BMI	0.000	1.064	(−0.020 to 0.019)	0.987		
NYHA class	0.154	1.243	(0.022 to 0.287)	< 0.05		
Diabetes	−0.191	1.114	(−0.385 to 0.004)	0.055		
**E/e'**					0.166	< 0.05
Constant term	1.750					
NLR	0.136	1.057	(0.014 to 0.258)	< 0.05		
Age	0.000	1.115	(−0.007 to 0.007)	0.914		
Female	0.167	1.089	(−0.012 to 0.347)	0.067		
BMI	0.016	1.060	(−0.002 to 0.035)	0.087		
NYHA class	0.112	1.223	(−0.015 to 0.239)	0.083		
Diabetes	0.015	1.166	(−0.175 to 0.205)	0.873		

The values were logarithmic transformed before analysis. Multivariate linear regression analysis was conducted in the HFpEF group (n = 172), with hs-CRP, NT-proBNP, and E/e' as dependent variables, respectively. The p < 0.05 was considered to indicate statistical significance. CI, confidence interval; VIF, variance inflation factors. Other abbreviations as in Table 1. P^*^, p-values of ANOVA test for individual models.

### Correlations Between Neutrophil Activation and Systemic Inflammation

The circulating levels of pro-inflammatory biomarkers were further examined in HFpEF patients and non-HF control individuals (Table 4, Supplementary Table 2). Multiple pro-inflammatory biomarkers involved in systemic inflammation, such as TNFα, IL-1β, IL-6, IL-10, and sICAM-1 (6, 7), were substantially increased in HFpEF patients. Meanwhile, compared to non-HF controls, HFpEF patients had a higher level of MMP9, a serological marker of collagen turnover that predicts diastolic dysfunction and incidence of HFpEF (19). We also found serum level of NE, one of the neutrophil-derived serine proteases released upon neutrophil activation and degranulation (20), was significantly elevated in the HFpEF group (Table 4). The circulating level of NE correlated well with multiple inflammatory biomarkers, including TNFα, IL-1β, IL-6, and sICAM-1. Meanwhile, these inflammatory biomarkers had a significant correlation with the mitral E/e' ratio. Finally, a correlation between NE and the E/e' ratio (*r* = 0.562, *p* < 0.01) was observed in HFpEF patients. By contrast, although MMP9 showed a correlation trend with both NE and E/e' ratio, it did not reach a statistical significance (Table 5).

**Table 4:** The concentration of inflammatory biomarkers in the circulation of non-HF control individuals and HFpEF patients.

	**Non-HF**	**HFpEF**	***p*-Value**
	**(*n =* 42)**	**(*n =* 30)**	
**Laboratory**			
TNFα, pg/ml	10.20 (2.42, 12.74)	12.38 (5.75, 19.70)	< 0.05
IL-1β, pg/ml	3.46 (2.53, 4.97)	17.93 (5.45, 21.93)	< 0.05
IL-6, pg/ml	1.72 (1.09, 2.18)	3.79 (1.25, 7.53)	< 0.05
IL-10, pg/ml	0.26 (0.12, 0.35)	0.38 (0.22, 1.16)	< 0.05
MMP9, ng/ml	270.1 (180.2, 390.6)	621.3 (302.5, 915.4)	< 0.05
sICAM, ng/ml	401.5 (257.2, 536.8)	583.2 (337.6, 721.5)	< 0.05
NE, ng/ml	83.28 (60.5, 134.3)	121.5 (90.6, 374.0)	< 0.05

*Data are given as median (IQR). Mann-Whitney test for unpaired observations was applied, and p < 0.05 was considered to indicate statistical significance*.

*IL, interleukin; MMP9, matrix metalloproteinase 9; NE, neutrophil elastase; sICAM-1, soluble intercellular adhesion molecule-1; TNFα, tumor necrosis factor alpha*.

**Table 5:** The interrelation between neutrophil elastase, inflammatory biomarkers, and mitral E/e' ratio.

		**TNFα**	**IL-1β**	**IL-6**	**IL-10**	**MMP9**	**sICAM-1**	**NE**	**E/e'**
**HFpEF (*****n=*** **30)**									
**NE**	***r***	0.735	0.636	0.809	0.250	0.302	0.546	—	0.562
	***p***	< 0.001	< 0.001	< 0.001	0.219	0.134	< 0.01	—	< 0.01
**E/e'**	***r***	0.673	0.547	0.670	0.171	0.235	0.561	0.562	—
	***p***	< 0.01	< 0.01	< 0.001	0.405	0.248	< 0.01	< 0.01	—

*The values were logarithmic transformed before analysis. Spearman's coefficients were computed to describe the correlation between the two variables. The significant differences were accepted when the p < 0.05, all abbreviations as in Tables 1, 4*.

### The Transcriptomic Characteristics of Neutrophils of HFpEF Patients

**Figure 2:**
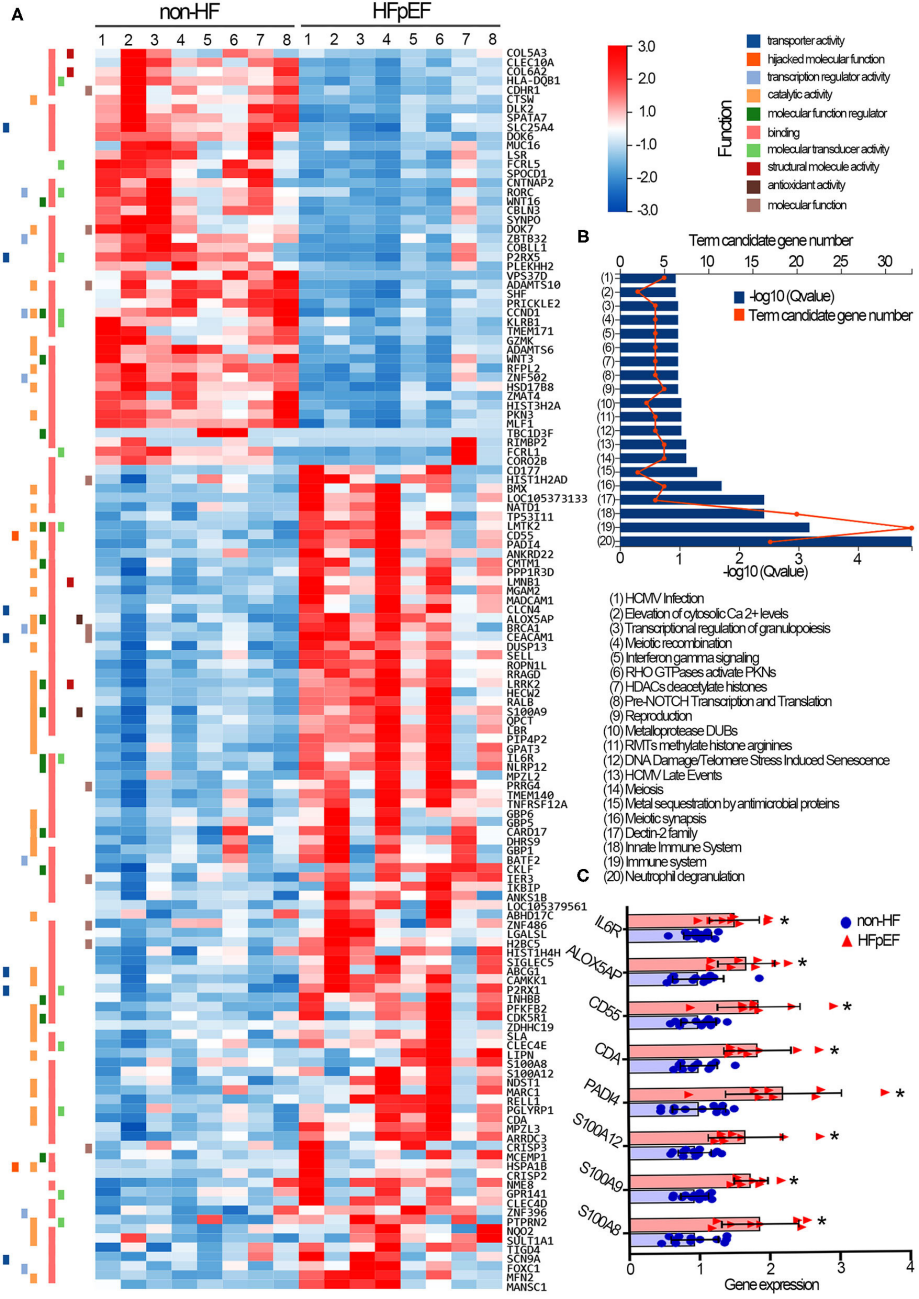
Transcriptomic profile of circulating neutrophils collected from non-HF control individuals and HFpEF patients. **(A)** The representative heatmap of the 134 genes that were differentially expressed in neutrophils of non-HF control individuals and HFpEF patients. Red indicates relative gene up-regulation, and blue indicates relative gene down-regulation. **(B)** The Reactome enrichment analysis was performed to characterize signaling pathways that genes participate. **(C)** Gene expression levels of *S100A8, S100A9, S100A12, PADI4, CDA, CD55, ALOX5AP*, and *IL6R* were compared in neutrophils of two groups. Fold change was calculated for comparison and was presented with means and SD. *p < 0.05, compared with non-HF control individuals. Non-HF, non-heart failure controls (*n* = 12); HFpEF, heart failure with preserved ejection fraction (*n* = 8).

We further characterized the transcriptional plasticity of neutrophils collected from non-HF controls and HFpEF patients. A total of 19,813 genes were successfully identified by RNA-sequencing. Among them, 134 genes were filtered with a significantly differential expression between the two groups (Supplementary Table 3). Concretely, compared with neutrophils of the non-HF control group, there were 89 transcripts significantly increased, whereas 45 transcripts decreased in neutrophils of HFpEF patients. The representative gene expression profile in the form of a heatmap was generated (Figure 2A). The Reactome annotation classification was subsequently performed to enrich signaling pathways that genes participate. The most significant enrichments were found in signaling pathways relating to neutrophil degranulation (17 genes), immune system (33 genes), and innate immune system (20 genes) (Figure 2B). Importantly, we found all 17 genes involved in neutrophil degranulation were significantly up-regulated in the HFpEF group. In particular, the gene expression of *S100A8* and *S100A9*, both of them encoding small calcium-binding protein S100A8/A9 complex, were significantly increased in neutrophils of HFpEF patients (Figure 2C). S100A8/A9 complex triggers leukocyte degranulation by promoting protein synthesis of leukotriene B4 (21) or by mechanisms dependent on p38 and JNK (22). In addition, HFpEF patients showed transcriptional up-regulation of *S100A12* in circulating neutrophils. The S100A12 protein has been proved to mobilize neutrophils from bone marrow and activate the adhesion and migration of neutrophils toward inflammatory sites (23). Compared with the non-HF controls, neutrophils of the HFpEF patients also had much higher *PADI4* gene expression that encodes the peptidyl arginine deiminase 4 (PAD4), a protein that critically regulates chromatin de-condensation and NETs formation (Figure 2C) (24). The gene-level of *CD55*, encoding a glycoprotein involved in the complement cascade regulation, was elevated in the HFpEF patients' neutrophils. On the resting neutrophil surface, the CD55 protein level is low, but that is highly expressed upon neutrophil activation (25). Finally, multiple transcripts (*CDA, ALOX5AP, IL6R*) with relatively high abundance in neutrophils were up-regulated in the HFpEF group as well. However, their pathophysiological relevance with cellular activation remains obscure yet.

## Discussion

The main findings from the present HFpEF study were as follows: (1) the patients in Southeast China were lean and who had comorbidity burden and worse cardiac structure and function; (2) the high NLR was predictive to HFpEF and independently associated with hs-CRP, NT-proBNP, and mitral E/e' ratio; (3) the heightened serum NE levels correlated with the systemic inflammation and mitral E/e' ratio in HFpEF patients; (4) multiple molecules that drive neutrophil degranulation and inflammation were transcriptionally up-regulated in neutrophils of HFpEF patients.

The clinical HFpEF is frequently bound with a broad of comorbidities (1, 4, 26). Among those comorbidities, obesity is highly prevalent in Western patients. The obese patients (BMI>30 Kg/m^2^) exhibit comorbidity-driven microvascular inflammation, HF severity, and fibrosis (27). By comparison, recent epidemiologic studies suggest a unique lean phenotype of HFpEF in Asia. The lean HFpEF patients have a high prevalence of diabetes and worse quality of life (28). Our HFpEF cohorts were lean, with an average BMI below 30 Kg/m^2^, in line with findings from the China HF registry (29). Besides the high prevalence of comorbidities, such as hypertension (71.5%), arrhythmia (64.5%), and coronary artery diseases (51.7%), diabetes was also found to be relatively common in HFpEF patients (33.7%) than that in non-HF cohorts (17.9%). In terms of pharmacological therapies, to date, the evidence-based therapies for HFpEF are scant. As such, current management of HFpEF is primarily directed toward associated cardiovascular comorbidities and control of hypervolemia (26). We found most HFpEF patients were on diuretics, ACEI/ARBs, and beta-blocker therapies. Although the evidence that beta-blockers improve symptoms in HFpEF patients is lacking, these medications were frequently prescribed to our HFpEF patients (87.2%) in order to lower cardiac oxygen demand and prolong diastolic filling time. The high rate of beta-blocker use is also found in HFpEF patients from the Asian-HF registry (78.9%), CHECK-HF registry (78%), and EMPEROR-Preserved trial (86%) (28, 30, 31). There has been compelling evidence to support the prominent role of inflammation in the pathogenesis and progression of HFpEF (5–7, 32, 33). Mechanically, pro-inflammatory molecules augment oxidative stress, impair nitric oxide bioavailability, reduce cyclic guanosine monophosphate activity but raise cardiomyocyte hypertrophy and passive stiffness. Microvascular inflammation drives the proliferation and activation of myofibroblasts. Abnormal extracellular matrix turnover triggered by pro-inflammatory molecules contributes to cardiac interstitial fibrosis (5, 8, 34). Our lean HFpEF patients had high levels of inflammatory biomarkers in circulation, including hs-CRP, TNFα, IL-1β, IL-6, IL-10, and sICAM-10. At present, identifying which organ(s) or cells are inflamed in patients is still tricky.

Systemic inflammation and immune cell homeostasis are two interlinked processes that constantly emphasize each other. The important role of monocytes and macrophages in cardiovascular inflammation has been historically appreciated (35). By contrast, neutrophils have been neglected in the context of cardiovascular research for a long time. Currently, our understanding of the pleiotropic roles of neutrophils in chronic inflammation has been advanced (9, 36). Aberrant neutrophils in circulation can stratify the risk of patients hospitalized with HFpEF or predict the poor prognosis of patients (11–13). In cardiac specimens of both HFpEF patients and animals, the neutrophil infiltration is found to be associated with inflammatory and fibrotic damages that result in LV stiffness (14, 15). In lean HFpEF patients, we observed an apparent rise in NLR ratio and serum levels of NE. Multivariate regression analysis revealed a clear association between the NLR and hs-CRP, NT-proBNP, and mitral E/e' ratio. Moreover, NLR was likely to be predictive of the increased risk of HFpEF. Of note, such associations were independent of the effect of age, gender, BMI, NYHA class, and diabetes. Meanwhile, the elevated serum NE levels in HFpEF patients significantly correlated with multiple pro-inflammatory biomarkers. Both of them also displayed a close correlation with the mitral E/e' ratio of patients. These interrelations collectively indicated the pathological potential of activated neutrophils in aggravating systemic inflammation and diastolic dysfunction of HFpEF patients. To date, the pathophysiological mechanisms responsible for neutrophils' detrimental effects on heart tissues remain to be elucidated yet. In the future study, it is of significant interest to resolve this doubt by investigating the cardiac phenotypes and systemic inflammation levels in our lean HFpEF mice after the genetic depletion of neutrophils (15).

So far, a number of risk factors have been proposed to drive granulopoiesis, including metabolic alternations (hypercholesterolemia and hyperglycemia), inflammasome pathways, aging, stress, and disturbed lifestyle (9). We noted that the distribution of age and gender was comparable between HFpEF patients and non-HF control individuals. The comorbid hyperlipidemia was neither prevalent in HFpEF patients (11.6%). By comparison, diabetes was found to be relatively common in HFpEF patients. Hyperglycemia directly induces proliferation and expansion of bone marrow myeloid progenitors (37, 38). Under chronic inflammation, some cytokines function as critical pro-inflammatory “emergency” signals to drive myeloid differentiation. IL-1β directly accelerates myeloid differentiation of hematopoietic stem cells *via* precocious activation of a PU.1-dependent gene program (39). Myocardial infarction results in rapid recruitment of neutrophils to the infarct. The infiltrated neutrophils release IL-1β, which may contribute to the cytokine pool. As a consequence, IL-1β acts with hematopoietic progenitor cells in the bone marrow and further stimulates granulopoiesis in a cell-autonomous manner (40). Alternatively, other inflamed tissues or cells may produce cytokine that accelerates myelopoiesis and neutrophil production, leading to neutrophil recruitment in heart tissues. However, the risk factors that drive neutrophilia in lean HFpEF patients remain unknown yet.

Although neutrophils are traditionally considered to be transcriptionally silent, the transcriptional plasticity of neutrophils upon sterile stimulation and microbial insults has been unraveled (41). We further found the transcriptional signatures of neutrophils of HFpEF patients were distinctive to that of non-HF control individuals. Beyond our expectation, circulating neutrophils from HFpEF patients do not show robust transcriptomic changes of the classical pro-inflammatory cytokine found in primed neutrophils *in vitro* (42). However, we noted multiple molecules that drive neutrophil degranulation and inflammation were transcriptionally up-regulated in neutrophils of HFpEF patients. Of note, among 134 genes with differential expression, all genes enriched in the neutrophil degranulation pathway were up-regulated in HFpEF patients' neutrophils, consistent with an increased level of neutrophil-derived NE in patients' blood. Moreover, genes encoding a small calcium-binding protein family (S100A8/A9/A12) were transcriptionally up-regulated in neutrophils of HFpEF patients. S100A8/A9 functions as neutrophil-derived alarmins that can activate CD11b and induce neutrophil adhesion to fibrinogen, leading to neutrophil migration to inflammatory sites (43). Hyperglycemia can increase the release of S100A8/S100A9 from neutrophils, and this protein complex interacts with the receptor for advanced glycation end products on myeloid progenitor cells and enhance myelopoiesis (37, 38). In infarct myocardial tissues, S100A8/S100A9 released from neutrophils can bind to Toll-like receptor (TLR) 4 and prime the nod-like receptor family pyrin domain-containing 3 inflammasome in naive neutrophils, resulting in IL-1β-driven granulopoiesis (40). It is particularly worth noting that *PADI4* is up-regulated substantially in neutrophils of HFpEF patients. PAD4 critically regulates chromatin de-condensation and NETs formation (24). The pathogenic potential of NETs in cardiovascular inflammation has so far been well-documented (44). NETs license macrophages to turn on transcriptional regulation of IL-6 and pro-IL-1β via TLR2/4 in atherosclerosis (45). NETs stimulate human lung fibroblasts to a myofibroblast with elevated α-smooth muscle actin expression (46) and mediates extracellular matrix remodeling (47). The cytotoxic histone and deoxyribonucleic acid bound to NETs induces organ fibrosis in aged mice (48). However, in clinical HFpEF patients, it is still difficult to determine whether neutrophils with high expression of *PADI4* are prone to form NETs in failing heart tissues. In lean HFpEF mouse hearts, we observed the presence of NETs and increased PAD4 protein levels, which was paralleled with cardiac inflammation and fibrosis (15). Our ongoing study further demonstrated neutrophils from lean HFpEF mice were prone to form NETs. The NETs-containing media significantly enhanced alpha-smooth muscle actin expression in co-cultured myocardial fibroblasts, suggesting a pro-fibrotic action of NETs (unpublished data).

Given the significant roles of neutrophils in cardiovascular inflammation, the specific intervention of neutrophils may open the door for the development of a novel therapeutic strategy. Interestingly, metformin, a drug representing a worldwide cornerstone in anti-diabetes therapy, can exert inflammation-inhibitory effects independently from glucose control (49). Metformin can inhibit NETs *in vitro* (50), decrease NLR in the diabetic population, and suppress plasma cytokine levels in the non-diabetic heart failure cohort (51). It is recently reported that, in patients who are infected with coronavirus disease 2019, metformin users have a lower level of neutrophil counts but a higher level of lymphocyte counts in the blood. Meanwhile, serum inflammatory factors (CRP, IL-6, TNF-α) and cardiac injury indicators (NT-proBNP) are marked lower in the metformin group (52). Therefore, we think repurposing metformin to inflammation-driven chronic HFpEF would be an active field investigated in the future.

## Study Limitations

Several limitations should be considered when interpreting the results of the present study. This was a small single cohort study. There was, therefore, a potential lack of power. The roles of neutrophils contributing to systemic or myocardial inflammation should be investigated in larger HFpEF cohorts. In addition, our investigation was not exploratory but based on the published hypothesis that inflammation is a critical pathogenic stimulus in HFpEF. We observed several correlations between neutrophil activation, systemic inflammation, and ventricular functional impairment in HFpEF patients. However, the tissue or cellular source of inflammatory molecules and their interrelation with neutrophilia in patients remain uncertain yet. Given the limitation to obtain heart specimens from clinical patients, the mechanisms by which neutrophils and NETs impair cardiac function need to be addressed by more intensive animal and *in vitro* studies.

## Conclusion

The high NLR coupled with transcriptional activation of neutrophils correlates with systemic inflammation and functional impairment in HFpEF patients, which may suggest a causative role of neutrophils in the pathogenesis of the disease.

## Data Availability Statement

The data used for the transcriptomic analysis were deposited in the NCBI Sequence Read Archive (SRA) database. The data are accessible via the SRA accession: PRJNA717666.

## Ethics Statement

The studies involving human participants were reviewed and approved by Research Ethics Committees of Shenzhen Second People's Hospital. The patients/participants provided their written informed consent to participate in this study.

## Author Contributions

BB, HC, and YX were responsible for the study design and manuscript writing. BB, MC, LJ, and JX contributed to the acquisition and analysis of the data. All authors gave final approval and agreed to be accountable for all aspects of work to ensure integrity and accuracy.

## Conflict of Interest

The authors declare that the research was conducted in the absence of any commercial or financial relationships that could be construed as a potential conflict of interest.
