# Supplementary material for: High Neutrophil to Lymphocyte Ratio and Its Gene Signatures Correlate With Diastolic Dysfunction in Heart Failure With Preserved Ejection Fraction
Source: Front Cardiovasc Med. 2021 Jun 24;8:614757. doi: 10.3389/fcvm.2021.614757 (PMC8263935; doi:10.3389/fcvm.2021.614757)
Supplement: Supplementary file 1 [file Data_Sheet_1.PDF]

Supplementary table 1: The correlations between NLR and parameters of cardiac structure or function of HFpEF patients.

**HFpEF** (n=172)												
NLR												
	r^a	p^a	r^b	p^b	r^c	p^c	r^d	p^d	r^e	p^e	r^f	p^f
LV mass	-0.058	0.454	-0.056	0.460	-0.061	0.432	-0.035	0.699	-0.063	0.416	-0.053	0.496
LA diameter	-0.037	0.632	-0.030	0.705	-0.026	0.735	-0.044	0.633	-0.064	0.416	-0.050	0.527
E/e'	0.278	**<0.01**	0.289	**<0.01**	0.291	**<0.01**	0.224	**<0.05**	0.270	**<0.01**	0.272	**<0.01**
NT-proBNP	0.341	**<0.001**	0.324	**<0.001**	0.340	**<0.001**	0.327	**<0.001**	0.314	**<0.001**	0.350	**<0.001**
hs-CRP	0.449	**<0.001**	0.451	**<0.001**	0.443	**<0.001**	0.413	**<0.001**	0.450	**<0.001**	0.447	**<0.001**

The values were logarithmic transformed before analysis. Partial Pearson correlations were computed to examine the relationship between variables of interest, without adjustment (***a***) or with adjustment for age (***b***), gender (***c***), BMI (***d***), NYHA class (***e***), and diabetes (***f***). The significant differences were accepted when the *p*-value was less than 0.05; other abbreviations as in Table 1.

Supplementary table 2: The clinical characteristics of non-HF control individuals and HFpEF patients for biomarker assay.

	**Non-HF** (n=42)	**HFpEF** (n=30)	***p*-Value**
**Demographic characteristics**			
Age	69.6±8.6	70.3±7.2	0.15
Female	22(52.3)	16(53.3)	0.99
BMI, kg/m^2	24.8±2.6	25.4±1.7	0.35
Heart rate, beats/min	74.3±9.7	85.4±10.3	**<0.05**
Systolic BP, mm Hg	135.9±16.1	139.6±15.8	0.65
Diastolic BP, mm Hg	81.4±13.2	80.8±14.7	0.73
**Medical history**			
NYHA functional class			**<0.05**
I	2(4.7)	2(6.7)	
II	0(0)	4(13.3)	
III	0(0)	16(53.3)	
IV	0(0)	8(26.7)	
Hypertension	42(100)	23(76.7)	**<0.05**
Diabetes mellitus	5(11.9)	12(40.0)	**<0.05**
Hyperlipidemia	6(14.2)	5(16.7)	0.99
Arrhythmia	6(14.2)	21(70)	**<0.05**
Coronary vascular disease	2(4.8)	18(60)	**<0.05**
**Medication use**			
Antiplatelet therapy	31(73.8)	22(73.3)	0.99
Beta-blockers	25(59.5)	19(63.3)	0.99
Calcium-channel blockers	22(52.3)	10(33.3)	**<0.05**
Diuretics	1(2.3)	26(86.7)	**<0.05**
ACE inhibitors or ARBs	21(50)	21(70)	**<0.05**
Statins	32(76.2)	25(83.3)	0.56
**Echocardiography**			
LV mass, g	155(139,172)	270(246,297)	**<0.05**
LVEF, %	67(61,71)	66(61,68)	0.22
E/e'	9.7(7.5,11.8)	16.0(13.9,20.8)	**<0.05**
LA diameter, cm	3.1(2.9,3.3)	4.0(3.5,4.2)	**<0.05**
**Laboratory**			
NT-proBNP, pg/ml	78(59,105)	2614(1236,5585)	**<0.05**
hs-CTnI, ng/ml	0.01(0.01,0.01)	0.01(0.01,0.01)	0.44
Creatinine, μmol/L	75.3(64.1,89.3)	76.1(61.0,90.3)	0.88
Total triglyceride, mmol/L	1.36(1.07,2.00)	1.16(1.03,1.78)	0.93

Total cholesterol, mmol/L	4.68(3.77,5.39)	4.00(3.21,4.78)	**<0.05**
LDL-C, mmol/L	3.00(2.48,3.60)	2.33(1.74,3.29)	**<0.05**
HDL-C, mmol/L	1.12(0.96,1.36)	1.02(0.78,1.22)	0.07
Fasting Glucose, mmol/L	5.28(4.66,5.81)	5.66(4.71,6.57)	0.26
HbA1c,%	5.8(5.5,6.2)	6.1(5.8,6.9)	**<0.05**
hs-CRP, mg/L	0.4(0.21,1.0)	10.2(2.7,10.6)	**<0.05**
**Hematological parameters**			
WBC count , 10^9/L	6.45(5.38,7.52)	6.29(4.82,8.25)	0.69
RBC count , 10^{12}/L	4.62(4.17,5.21)	4.54(3.61,4.88)	0.11
Platelet count, 10^9/L	230(198,263)	215(175,280)	0.45
Hemoglobin, g/L	139(128,146)	133(118,148)	0.18
Neutrophil, 10^9/L	3.81(3.04,4.51)	4.53(3.00,5.91)	**<0.05**
Lymphocyte, 10^9/L	1.87(1.48,1.88)	1.38(1.13,1.59)	**<0.05**
Monocyte, 10^9/L	0.41(0.36,0.55)	0.41(0.36,0.50)	0.75
NLR	2.00(1.82,2.56)	3.37(2.85,4.73)	**<0.05**

Data are given as mean (SD), median (IQR), or number (percent), as appropriate.

Depending on the types of data, Mann-Whitney test or Fisher exact test for unpaired observations were applied, and *p*-values less than 0.05 were considered to indicate statistical significance.

ACEI, angiotensin-converting enzyme inhibitors; ARBs, receptor blockers; BMI, body mass index; BP, blood pressure; HbA1c, hemoglobin A1c; HDL-C, high-density lipoprotein cholesterol; hs-Tnl, high-sensitive cardiac troponin I; hs-CRP, high-sensitivity C-reactive protein; LA, left atrial; LDL-C, low-density lipoprotein cholesterol; LVEF, left ventricular ejection fraction; LV mass, left ventricular mass; NT-proBNP, N-terminal prohormone of brain natriuretic peptide; NYHA, New York Heart Association; RBC, red blood cell; WBC, white blood cell; NLR=neutrophil count to lymphocyte count.

Supplementary table 3: Transcriptomic profile of circulating neutrophils collected from non-HF control individuals and HFpEF patients.

NCBI Gene ID	Gene Symbol	HFpEF *vs* . non-HF
10170	DHRS9	Up-regulation
10205	MPZL2	
10321	CRISP3	
105373133	LOC105373133	
105379561	LOC105379561	
113540	CMTM1	
115362	GBP5	
116071	BATF2	
1183	CLCN4	
118932	ANKRD22	
120892	LRRK2	
121457	IKBIP	
131540	ZDHHC19	
1604	CD55	
163351	GBP6	
196264	MPZL3	
199675	MCEMP1	
201798	TIGD4	
22853	LMTK2	
2296	FOXC1	
23569	PADI4	
241	ALOX5AP	
252884	ZNF396	
256302	NATD1	
25797	QPCT	
26253	CLEC4E	
2633	GBP1	
29094	LGALSL	
3013	H2AC7	
3017	H2BC5	
3304	HSPA1B	
3340	NDST1	
338339	CLEC4D	
353345	GPR141	
3570	IL6R	
3625	INHBB	

3930	LBR	Up-regulation
4001	LMNB1	
440068	CARD17	
4835	NQO2	
5023	P2RX1	
51192	CKLF	
51207	DUSP13	
51314	NME8	
51330	TNFRSF12A	
5208	PFKFB2	
54682	MANSC1	
5509	PPP1R3D	
55281	TMEM140	
55529	PIP4P2	
56899	ANKS1B	
57126	CD177	
57520	HECW2	
57561	ARRDC3	
5799	PTPRN2	
58489	ABHD17C	
58528	RRAGD	
5899	RALB	
6279	S100A8	
6280	S100A9	
6283	S100A12	
6335	SCN9A	
634	CEACAM1	
6402	SELL	
643418	LIPN	
64757	MTARC1	
6503	SLA	
660	BMX	
672	BRCA1	
6817	SULT1A1	
7180	CRISP2	
768211	RELL1	

79056	PRRG4	Up-regulation
8174	MADCAM1	
8365	H4C8	
83853	ROPN1L	
84254	CAMKK1	
84803	GPAT3	
8778	SIGLEC5	
8851	CDK5R1	
8870	IER3	
8993	PGLYRP1	
90649	ZNF486	
91662	NLRP12	
93432	MGAM2	
9537	TP53I11	
9619	ABCG1	
978	CDA	
9927	MFN2	
10391	CORO2B	Down-regulation
10462	CLEC10A	
10739	RFPL2	
11174	ADAMTS6	
11346	SYNPO	
115350	FCRL1	
1292	COL6A2	
130271	PLEKHH2	
134285	TMEM171	
1521	CTSW	
155382	VPS37D	
166336	PRICKLE2	
220164	DOK6	
22837	COBLL1	
23504	RIMBP2	
26047	CNTNAP2	
27033	ZBTB32	
285489	DOK7	

291	SLC25A4	Down-regulation
29941	PKN3	
3003	GZMK	
3119	HLA-DQB1	
3820	KLRB1	
4291	MLF1	
5026	P2RX5	
50509	COL5A3	
51384	WNT16	
51599	LSR	
55812	SPATA7	
595	CCND1	
6097	RORC	
643866	CBLN3	
65989	DLK2	
7473	WNT3	
7923	HSD17B8	
79698	ZMAT4	
81794	ADAMTS10	
83416	FCRL5	
84218	TBC1D3F	
90525	SHF	
90853	SPOCD1	
91392	ZNF502	
92211	CDHR1	
92815	H2AW	
94025	MUC16	
